# Morphology and ASAP analysis of the important zoonotic nematode parasite *Baylisascaris procyonis* (Stefahski and Zarnowski, 1951), with molecular phylogenetic relationships of *Baylisascaris* species (Nematoda: Ascaridida)

**DOI:** 10.1017/S0031182023001312

**Published:** 2024-02

**Authors:** Xiao-Hong Gu, Hui-Xia Chen, Jun-Jie Hu, Liang Li

**Affiliations:** 1 Hebei Key Laboratory of Animal Physiology, Biochemistry and Molecular Biology; Hebei Collaborative Innovation Centre for Eco-Environment; College of Life Sciences, Hebei Normal University, 050024 Shijiazhuang, Hebei Province, People's Republic of China; 2 Hebei Research Centre of the Basic Discipline Cell Biology, Ministry of Education Key Laboratory of Molecular and Cellular Biology, 050024 Shijiazhuang, Hebei Province, People's Republic of China; 3 School of Ecology and Environmental Sciences, Yunnan University, 650091, Kunming, People's Republic of China

**Keywords:** Ascaridida, *Baylisascaris*, genetic data, morphology, phylogeny, species delimitation, zoonotic nematode

## Abstract

Species of *Baylisascaris* (Nematoda: Ascarididae) are of great veterinary and zoonotic significance, owing to cause Baylisascariosis or Baylisascariasis in wildlife, captive animals and humans. However, the phylogenetic relationships of the current 10 *Baylisascaris* species remain unclear. Moreover, our current knowledge of the detailed morphology and morphometrics of the important zoonotic species *B. procyonis* is still insufficient. The taxonomical status of *B. procyonis* and *B. columnaris* remains under debate. In the present study, the detailed morphology of *B. procyonis* was studied using light and scanning electron microscopy based on newly collected specimens from the raccoon *Procyon lotor* (Linnaeus) in China. The results of the ASAP analysis and Bayesian inference (BI) using the 28S, ITS, *cox*1 and *cox*2 genetic markers did not support that *B. procyonis* and *B. columnaris* represent two distinct species. Integrative morphological and molecular assessment challenged the validity of *B. procyonis*, and suggested that *B. procyonis* seems to represent a synonym of *B. columnaris.* Molecular phylogenetic results indicated that the species of *Baylisascaris* were grouped into 4 clades according to their host specificity. The present study provided new insights into the taxonomic status of *B. procyonis* and preliminarily clarified the phylogenetic relationships of *Baylisascaris* species.

## Graphical abstract

**Figure:**
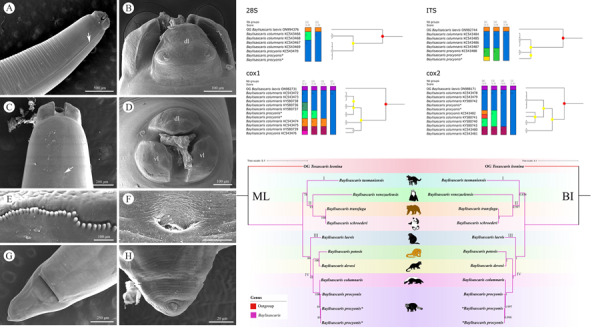

## Introduction

Species of *Baylisascaris* (Nematoda: Ascarididae) mainly occur in the digestive tract of carnivores and omnivores worldwide (Sprent, 1968, 1970; Bauer, 2013), which are of great veterinary and zoonotic significance, owing to cause Baylisascariosis or Baylisascariasis in wildlife, captive animals and humans (Sato *et al.*, 2004; Bauer, 2013; Graeff-Teixeira *et al.*, 2016). The life cycle of *Baylisascaris* spp. seems to be heteroxenous, and the second-stage larvae in eggs are considered to be the infective stage (Bauer, 2013). When infective eggs are ingested by small mammals including humans, the second-stage larvae hatch from eggs, then penetrate the intestine wall and migrate into various organs and tissues causing ocular larva migrans (OLM), visceral larva migrans (VLM) and neural larva migrans (NLM) (Gavin *et al.*, 2005; Bauer, 2013; Graeff-Teixeira *et al.*, 2016).

According to Sprent (1968) and Bauer (2013), 9 species were assigned to *Baylisascaris*, namely *B. transfuga* (Rudolphi, 1819), *B. laevis* (Leidy, 1856), *B. columnaris* (Leidy, 1856), *B. melis* (Gedoelst, 1920), *B. schroederi* (McIntosh, 1939), *B. procyonis* (Stefanski and Zarnowski, 1951), *B. devosi* (Sprent, 1952), *B. tasmaniensis* Sprent, 1970 and *B. ailuri* (Wu *et al.*, 1987). Later, *B. potosis* Tokiwa *et al.*, 2014 and *B. venezuelensis* Mata *et al.*, 2016 were described from Japan and Venezuela, respectively (Tokiwa *et al.*, 2014; Mata *et al.*, 2016). In addition, Li *et al.* (2016) treated *B. ailuri* as a synonym of *B. transfuga*. Consequently, the genus *Baylisascaris* currently includes 10 recognized species. Although the phylogenetic analyses of *Baylisascaris* species were investigated using different genetic data in some previous studies (Nadler, 1992; Nadler and Hudspeth, 1998, 2000; Xie *et al.*, 2011*a*, 2011*b*; Tokiwa *et al.*, 2014; Mata *et al.*, 2016; Camp *et al.*, 2018; Li *et al.*, 2018; Sharifdini *et al.*, 2021), the phylogenetic relationships of these 10 *Baylisascaris* species remain unclear, because all of the previous phylogenetic studies only included a limited number of species.

The raccoon roundworm *B. procyonis*, is widely distributed in North America, and can cause severe clinical disease in humans and animals, due to extensive larval migration through host tissues (Kazacos and Boyce, 1989; Kazacos, 2001; Gavin *et al.*, 2002, 2005; Graeff-Teixeira *et al.*, 2016). Although the morphological characters of *B. procyonis* have been reported by some previous studies (Stefanski and Zarnowski, 1951; Hartwich, 1962; Sprent, 1968; Overstreet, 1970; Kikuchi and Oshima, 1977; Kazacos and Turek, 1982; Snyder, 1989), our current knowledge of the detailed morphology and morphometrics of *B*. *procyonis* is still insufficient. Furthermore, in the genus *Baylisascaris*, *B. procyonis* is highly similar to *B. columnaris* morphologically and genetically. Some recent studies based on different genetic data considered that *B. procyonis* and *B. columnaris* are closely related, but distinct species (Franssen *et al.*, 2013; Choi *et al.*, 2017); however, the results of other molecular studies did not support the current species partition of the 2 species (Camp *et al.*, 2018). The hypothesis that *B. procyonis* and *B. columnaris* represent 2 separate species still needs to be further tested using different methods or based on different genetic data and broader samples collected from different localities.

In the present study, several adults of *B. procyonis* were collected from the raccoon *Procyon lotor* (Linnaeus) (Mammalia: Carnivora) in the Zoo of Kunming, Yunnan Province, China. The detailed morphology of *B. procyonis* was further studied using light and scanning electron microscopy. The ASAP (Assemble Species by Automatic Partitioning) analysis and Bayesian inference (BI) were employed for delimitation of *B. procyonis* and *B. columnaris* based on different nuclear [large ribosomal DNA (28S) and internal transcribed spacer (ITS)] and mitochondrial [cytochrome c oxidase subunit 1 (*cox*1) and 2 (*cox*2)] genetic markers. Moreover, to evaluate the evolutionary relationships of *Baylisascaris* species, phylogenetic analyses including the most comprehensive taxa sampling of *Baylisascaris* to date, were performed based on the ITS and 28S + ITS + *cox*1 + *cox*2 sequence data using maximum likelihood (ML) and Bayesian inference (BI) methods, respectively.

## Materials and methods

### Specimen collection and morphological study

Single *Procyon lotor* died naturally in the Zoo of Kunming, Yunnan Province, China, which was opportunistically dissected for parasites. Some nematode specimens were isolated from the small intestine of this raccoon. Nematodes were fixed and stored in 70% ethanol until the study. For light microscopy studies, nematodes were cleared in glycerine for examination using a Nikon® optical microscope. For scanning electron microscopy (s.e.m.), specimens were re-fixed in a 4% formaldehyde solution, post-fixed in 1% O_s_O_4_, dehydrated *via* an ethanol series (50, 70, 80, 90, 100, 100%) and acetone (100%), and then critical point dried. Samples were coated with gold at about 20 nm and examined using a Hitachi S-4800 scanning electron microscope at an accelerating voltage of 20 kV. Measurements (range, followed by mean in parentheses) are given in millimetres (mm) unless otherwise stated. Voucher specimens were deposited in College of Life Sciences, Hebei Normal University, Hebei Province, China.

### Molecular procedures

The mid-body parts of 2 randomly selected nematode specimens (1 male, 1 female) were used for molecular analysis. Genomic DNA from each sample was extracted using a Column Genomic DNA Isolation Kit (Shanghai Sangon, China) according to the manufacturer's instructions. The partial 28S region was amplified by PCR using the forward primer 28S-F (5′-AGC GGA GGA AAA GAA ACT AA −3′) and the reverse primer 28S-R (5′-ATC CGT GTT TCA AGA CGG G −3′) (Nadler and Hudspeth, 1998). The partial *cox*1 region was amplified by PCR using the forward primer COI-F (5′-TTT TTT GGT CAT CCT GAG GTT TAT −3′) and the reverse primer COI-R (5′-ACA TAA TGA AAA TGA CTA ACA AC −3′) (Lazarova *et al.*, 2006). The partial *cox*2 region was amplified by PCR using the forward primer COII-F (5′-AAT TTT AAT TGT AGT CTT TTG TTT GG −3′) and the reverse primer COII-R (5′-CTA TGA TTA GCA CCA CAA ATC −3′) (Nadler and Hudspeth, 1998). The partial ITS-1 region was amplified by PCR using the forward primer SS1 (5′- GTT TCC GTA GGT GAA CCT GCG −3′) and the reverse primer SS2R (5′- AGT GCT CAA TGT GTC TGC AA −3′). The ITS-2 region of nuclear rDNA was amplified by PCR using the forward primer NC13 (5′-ATC GAT GAA GAA CGC AGC-3′) and the reverse primer NC2 (reverse: 5′-TTA GTT TCT TTT CCT CCG CT-3′) (Zhu *et al.*, 2000).

All PCRs of nematodes were performed in 50 μL of PCR reaction buffer with 10 mm Tris–HCl at pH 8.4, 50 mm KCl, 3.0 mm MgCl_2_, 250 μm of each dNTP, 50 pmol of each primer and 1.5 U of Taq polymerase [Takara Biotechnology (Dalian) Co. Ltd., Japan] in a thermocycler (2720, Applied Biosystems) under the following conditions: the partial 28S region: 94°C, 5 min (initial denaturation), followed by 30 cycles of 94°C, 30 s (denaturation), 55°C, 30 s (annealing), 72°C, 70 s (extension), and a final extension of 72°C for 7 min; the partial ITS region: 94°C, 5 min (initial denaturation), followed by 30 cycles of 94°C, 30 s (denaturation), 55°C, 30 s (annealing), 72°C, 30 s (extension), and a final extension of 72°C for 7 min; the partial *cox*1 region: 94°C, 5 min (initial denaturation), followed by 30 cycles of 94°C, 30 s (denaturation), 55°C, 30 s (annealing), 72°C, 45 s (extension), and a final extension of 72°C for 7 min; the partial *cox*2 region: 95°C, 15 min (initial denaturation), followed by 35 cycles of 95°C, 60 s (denaturation), 45°C, 60 s (annealing), 72°C, 75 s (extension), and a final extension of 72°C for 7 min.

PCR products were checked on GoldView-stained 1.5% agarose gels and purified with Column PCR Product Purification Kit (Shanghai Sangon, China). Sequencing for each sample was carried out on both strands. Sequences were aligned using ClustalW2. The DNA sequences obtained herein were compared (using the algorithm BLASTn) with those available in the National Centre for Biotechnology Information (NCBI) database (http://www.ncbi.nlm.nih.gov). The 28S, ITS, *cox*1 and *cox*2 sequences data of *Baylisascaris procyonis* were deposited in the GenBank (http://www.ncbi.nlm.nih.gov).

### Species delimitation

The ASAP method (Puillandre *et al.*, 2021) and Bayesian inference were used for species delimitation of *Baylisascaris procyonis* and *B. columnaris* based on the 28S, ITS, *cox*1, and *cox*2 sequences, respectively. The BI trees were inferred using MrBayes 3.2.7 (Ronquist *et al.*, 2012) under the K81UF model for *cox*2, MTART + I + F for ITS, HKY + F + I for 28S and TRN for *cox*1 (two parallel runs, 1 000 000 generations). *Baylisascaris laevis* was chosen as the out-group. The ASAP analyses was conducted using the ASAP online server (https://bioinfo.mnhn.fr/abi/public/asap) under the Kimura (K80) ts/tv model. The results of ASAP with lowest scores were considered as the optimal group number, except the optimal results recommended by ASAP.

### Phylogenetic analyses

Phylogenetic analyses were performed based on the ITS and ITS + 28S + *cox*1 + *cox*2 sequence data using maximum likelihood (ML) inference with IQTREE v2.1.2 (Minh *et al.*, 2020) and Bayesian inference (BI) with MrBayes 3.2.7 (Ronquist *et al.*, 2012), respectively. *Toxascaris leonina* (Ascaridoidea: Ascarididae) was chosen as the out-group. The in-group included 9 *Baylisascaris* species. The species *B. melis* has no published sequence data; consequently, it was not included in the present phylogenetic analyses. The detailed information on *Baylisascaris* nematodes included in the present phylogenetic analyses is provided in Table 1.

**Table 1.** Species of *Baylisascaris* with detailed genetic information included in the phylogenetic analyses

Species	Host	Locality	GenBank ID				References
			ITS	28S	*cox*1	*cox*2	
*B. procyonis* (Stefanski and Zarnowski, 1951)	*Procyon lotor*	China	OR453333–OR453334	OR457646–OR457647	OR453235–OR453236	OR463271–OR463272	Present study
*B. transfuga* (Rudolphi, 1819)	*Ursus arctos; Melursus ursinus*	Netherlands	KC543489	KC543471	KC543477	KC543483	Franssen *et al.* (2013)
*B. laevis* (Leidy, 1856)	*Marmota vancouverensis; Urocitellus columbianus*	Canada; USA	ON982744	ON994376	ON982731	ON988170	Barrera *et al.* (2022)
*B. columnaris* (Leidy, 1856)	*Mephitis mephitis*	USA; Netherlands	KC543484	KC543466	KY580736	KC543478	Choi *et al.* (2017), Franssen *et al.* (2013)
*B. schroederi* (McIntosh, 1939)	*Ailuropoda melanoleuca*	China	MH030599	MG937777	MH795152	MH469667	Camp *et al.* (2018), Hoberg *et al.* (2018)
*B. procyonis* (Stefanski and Zarnowski, 1951)	*Procyon lotor*	Norway	KC543488	KC543470	KC543476	KC543482	Franssen *et al.* (2013)
*B. devosi* (Sprent, 1952)	*Pekania pennanti*	Canada	MH030598	MG937776	MH795151	MH469666	Camp *et al.* (2018), Hoberg *et al.* (2018)
*B. tasmaniensis* Sprent, 1970	*Sarcophilus harrisii*	Australia	MH030603	MG937781	MH795156	MH469671	Camp *et al.* (2018), Hoberg *et al.* (2018)
*B. potosis* Tokiwa *et al.* 2014	*Potos flavus*	Guyana	AB901104	AB893608	AB893609		Tokiwa *et al.* (2014)
*B. venezuelensis* Mata *et al.* 2016	*Tremarctos ornatus*	Venezuela	KX151726				Mata *et al.* (2016)

The nucleotide sequences were aligned in batches using MAFFT v7.313 under iterative refinement method of E-INS-I (Katoh and Standley, 2013), poorly aligned regions were excluded using BMGE v1.12 (*h* = 0.4) (Criscuolo and Gribaldo, 2010). Furthermore, partially ambiguous bases were manually inspected and removed. Substitution models were compared and selected according to the Bayesian Information Criterion by using ModelFinder (Kalyaanamoorthy *et al.*, 2017). The HKY + F + I and HKY + I model were identified as the optimal nucleotide substitution model for the ML and BI inference of ITS sequences. The partitioning schemes and the optimal nucleotide substitution model selected for each combination of partition for the ML and BI inference of ITS + 28S + *cox*1 + *cox*2 sequences are shown in Table 2. Reliabilities for maximum likelihood inference were tested using 1000 bootstrap replications and Bayesian Information Criterion analysis was run for 5 × 10^6^ MCMC generations.

**Table 2.** The partitioning schemes and the optimal model selected for each combination of partition for the ML and BI inference based on the ITS + 28S + *cox*1 + *cox*2 sequences

Subset	Best Model-BI	Best Model-ML	Number of sites	Partitioning schemes
1	K80 + I	K2P + FQ + I	1067	28S
2	HKY + I	K2Pu + F + I	1685	*cox*1; *cox*2
3	HKY + I	GTR + F + I + G4	927	ITS

In the ML tree, the bootstrap (BS) values ≥90 were considered to constitute strong nodal support, whereas BS values ≥70 and <90 were considered to constitute moderate nodal support. In the BI tree, the Bayesian posterior probabilities (BPP) values ≥0.90 were considered to constitute strong nodal support, whereas BPP values ≥0.70 and <0.90 were considered to constitute moderate nodal support. The BS values ≥70 and BPP values ≥0.70 were shown in the phylogenetic trees.

## Results

### Morphology of Baylisascaris procyonis

#### General

**Figure 1.**
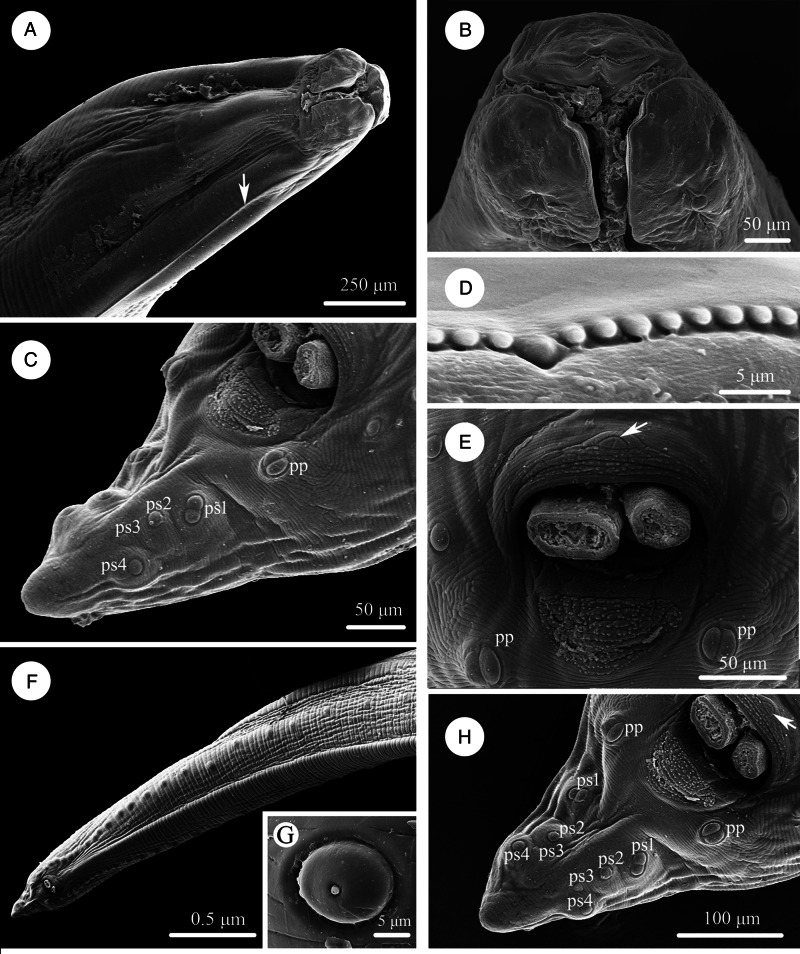
Scanning electron micrographs of *Baylisascaris procyonis* collected from *Procyon lotor* (Mammalia: Carnivora) in China, male. (A) Anterior part of body (lateral ala arrowed), lateral view. (B) Cephalic end, apical view. (C) Tail, ventrolateral view. (D) Magnified image of labial denticles. (E) Magnified image of cloacal area (medio-ventral precloacal papilla arrowed), ventral view. (F) Posterior end of body, ventral view. (G) Magnified image of postcloacal papillae. (H) Tail (medio-ventral precloacal papilla arrowed), ventral view. *Abbreviations*: pp, paracloacal double papillae; ps1, first pair of postcloacal double papillae; ps2-4, second to fourth pairs of postcloacal single papillae.

**Figure 2.**
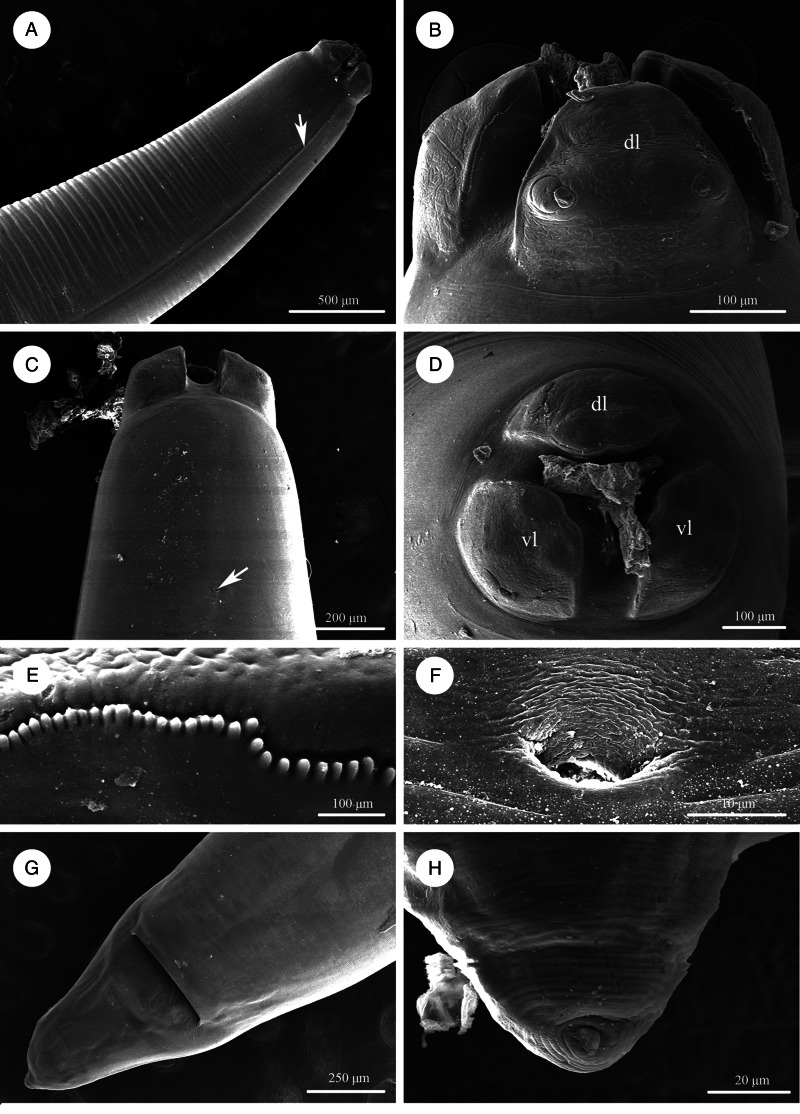
Scanning electron micrographs of *Baylisascaris procyonis* collected from *Procyon lotor* (Mammalia: Carnivora) in China, female. (A) Anterior part of body (lateral ala arrowed), lateral view. (B) Cephalic end, dorsal view. (C) Anterior part of body (excretory pore arrowed), ventral view. (D) Cephalic end, apical view. (E) Magnified image of labial denticles. (F) Magnified image of excretory pore. (G) Posterior end of body, ventral view. (H) Magnified image of tail tip. *Abbreviations:* dl: dorsal lip; vl: ventrolateral lip.

**Figure 3.**
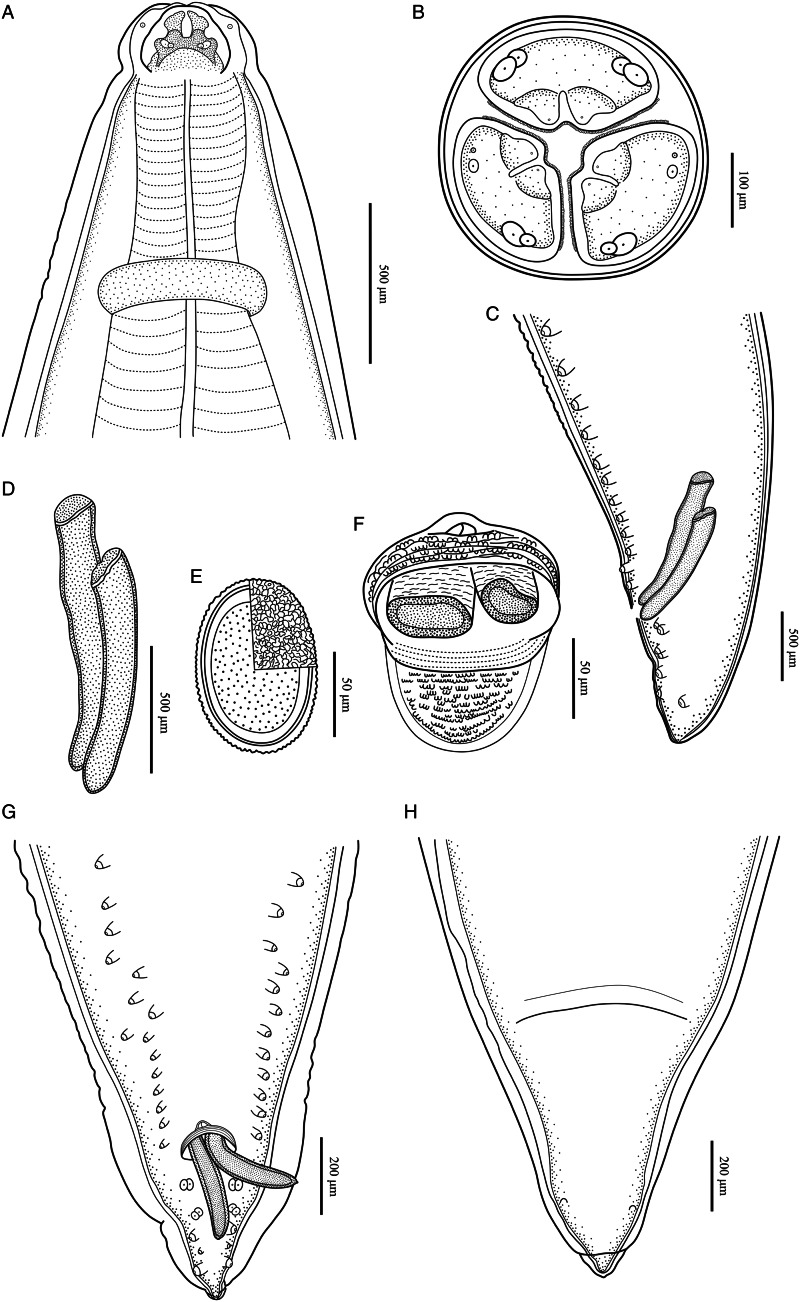
*Baylisascaris procyonis* collected from *Procyon lotor* (Mammalia: Carnivora) in China. (A) Anterior part of male, dorsal view. (B) Cephalic end of male, apical view. (C) Posterior end of male, lateral view. (D) Spicules. (E) Egg. (F) Magnified image of cloacal area, ventral view. (G) Posterior end of male, ventral view. (H) Posterior end of female, ventral view.

Large, whitish nematodes with finely transversely striated cuticle (Stefanski and Zarnowski, 1951) (Figures 1–3, Table 3). Maximum width of body at about mid-body. Cervical alae very narrow, starting from some distance from the base of ventrolateral lips and extending to anterior 1/4–1/2 of oesophageal length (Figs 1A, 2A); caudal alae absent. Cephalic extremity with 3 roughly trapezoid lips, postlabial grooves inconspicuous (Figs 1A, B; 2A–D, 3A, B). Dorsal lip with 1 pair of large double papillae; ventrolateral lips each with single double papilla, small papilla and amphid (Figs 2B, D, 3A, B). Each lip with distal ridge is armed with 140–150 small denticles (Figs 1B, D, 2B, D, E). Anterior margin of each lip with a small obtusely triangular medio-apical notch (Figs 1A, B, 2D, 3B). Interlabia absent. Oesophagus muscular, nearly cylindrical, distinctly broader posteriorly than anteriorly. Nerve-ring at about 20% of oesophageal length. Excretory pore just posterior to nerve-ring. Ventriculus, intestinal caecum and ventricular appendix absent. Tail of both sexes conical (Figs 1C, H; 2G, H, 3C, G, H).

**Table 3.** Morphometric comparisons of *Baylisascaris procyonis* (measurements in millimetres)

Characteristics	Present study		Stefanski and Zarnowski (1951)		Hartwich (1962)		Sprent (1968)		Overstreet (1970)			
	Male	Female	Male	Female	Male	Female	Male	Female	Male	Female	Male	Female
BL	66–72	99–152	62–76	90–122	62–77	90–200	90	200	57–99	87–160	57–119	49–189
OL	3.24–3.44	3.78–4.27	3.90	4.50	3.30–4.60	–	–	–	2.23–4.15	2.96–4.54	–	–
LSL	0.41–0.49	N/A	0.51–0.55	N/A	0.51–0.55	N/A	0.61	N/A	0.38–0.57	N/A	0.38–0.62	N/A
RSL	0.44–0.53	N/A	0.53–0.59	N/A	0.53–0.61	N/A	0.61	N/A	0.39–0.52	N/A	0.38–0.62	N/A
PRP	33–42 pairs	N/A	40–44 pairs	N/A	27–43 pair	N/A	43 pair	N/A	36–50 pairs	N/A	28–49 pairs	N/A
PDP	1 pair	N/A	1 pair	N/A	1 pair	N/A	–	N/A	1 pair	N/A	1 pair	N/A
PSP	1 pair double + 3 pairs single	N/A	1 pair double + 3 pairs single	N/A	1 pair double + 3 pairs single	N/A	–	N/A	1 pair double + 3 pairs single	N/A	1 pair double + 3 pairs single	N/A
VA	N/A	31–42	N/A	21–28	N/A	21–28	N/A	–	N/A	31.4–49.5	N/A	–
VA/BL (%)	N/A	27.6–31.3	N/A	–	N/A	–	N/A	–	N/A	30–36	N/A	28.0–36.0
TL	0.42–0.45	0.59–1.02	0.35–0.40	0.74–0.94	0.35–0.52	0.74–0.94	0.52	0.94	0.35–0.52	0.77–1.24	0.36–0.59	0.54–1.22
SE	N/A	0.07–0.08 × 0.05–0.07	N/A	0.08 × 0.05–0.06	N/A	0.07–0.08 × 0.05–0.07	N/A	0.08 × 0.07	N/A	0.07–0.08 × 0.05–0.07	N/A	–
OL/BL (%)	4.78–4.91	2.49–3.87	–	–	–	–	–	–	3.50–4.70	2.70–3.50	3.09–5.20	2.90–5.60
Host	*Procyon lotor*		*Procyon lotor*		*Procyon lotor*		*Procyon lotor*		*Potos flavus*		*Procyon lotor*	
Locality	China		Poland		?		USA, Canada		USA		USA	

BL, length of body; OL, length of oesophagus; RSL, length of right spicule; LSL, length of left spicule; PRP, number of precloacal papillae; PDP, number of paracloacal double papillae; PSP, number of postcloacal papillae; SE, size of egg; VA, distance from vulva to anterior extremity; TL, length of tail; SE, size of eggs. N/A represents ‘Not applicable’.

### Male (based on 2 mature specimens)

Body 66.0–72.0 (69.0) long, maximum width 1.70–1.76 (1.73). Dorsal and ventrolateral lips approximately equal in size, 0.23–0.28 (0.26) long, 0.30–0.32 (0.31) wide. Oesophagus 3.24–3.44 (3.34) long, 0.49–0.59 (0.54) in maximum width, representing 4.78–4.91 (4.85) % of body length. Nerve-ring and excretory pore 0.69–0.79 (0.74) and 0.93–1.15 (1.04) from anterior extremity, respectively. Cervical alae about 1.60 long. Posterior end of body curves ventrally. Spicules robust, ornamented with remarkable sculptures on surface, without alae, slightly unequal in length, distal end rounded, left spicule 0.44–0.53 (0.49) long, representing 0.61–0.80 (0.71) % body length, right spicule 0.41–0.49 (0.45) long and representing 0.57–0.74 (0.66) % body length, (Figs 1C, E, H, 3C, D, F, G). Gubernaculum absent. Caudal papillae 45–65 pairs in total, arranged as follows: 33–42 pairs precloacal, 1 pair paracloacal double papillae (slightly posterior to cloaca) and 4 pairs postcloacal (distal) (1st pair being double papillae, 3rd pair distinctly smaller than the others and 4th pair located sub-laterally) (the nomenclature of caudal papillae according to Fagerholm, 1991) (Figs 1C, E–H, 3C, G). Single medio-ventral precloacal papilla present (Figs 1E, H, 3F, G). Precloacal and postcloacal regions ornamented with rows of cuticular protuberance (commonly called ‘rugose area’ in previous studies). Precloacal ornamentation nearly meniscus-like, with 4–5 rows of cuticular protuberance; postcloacal ornamentation more or less semicircular, with 9–11 rows of cuticular protuberance (Figs 1C, E, H, 3F). Tail 0.42–0.45 (0.44) long, with rounded tip (Figs 1C, H, 3C, G).

### Female (based on 3 gravid specimens)

Body 99.0–152.0 (127.0) long; maximum width 1.76–2.44 (1.80). Dorsal and ventro-lateral lips approximately equal in size, 0.23–0.29 (0.26) long, 0.32–0.35 (0.33) wide. Oesophagus 3.78–4.27 (3.96) long, 0.40–0.98 (0.74) in maximum width, representing 2.49–3.87 (3.21) % of body length. Nerve-ring and excretory pore 0.60–0.94 (0.73) and 0.96–1.41 (1.11) from anterior extremity, respectively. Cervical alae about 0.98 long. Vulva slit-like, pre-equatorial, 31.0–42.0 (36.5) mm from anterior extremity, at 27.6–31.3 (29.5) % of body length. Vagina muscular, directed posteriorly from vulva. Eggs oval, with rough shell, 0.07–0.13 × 0.05–0.08 (*n* = 20) (Fig. 3E). Tail 0.59–1.02 (0.80) long, with small button-shaped tip (Figs 2G, H, 3H). Phasmids 0.17–0.26 (0.23) from tail tip.

### Species delimitation of *B. procyonis* and *B. columnaris*

**Figure 4.**
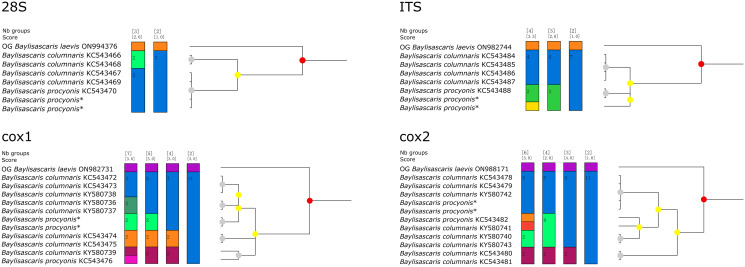
Assemble species by automatic partitioning (ASAP) analyses of *Baylisascaris procyonis* and *B. columnaris* based on 4 different nuclear and mitochondrial genetic markers. Abbreviations: *cox*1, cytochrome c oxidase subunit I; *cox*2, cytochrome c oxidase subunit II; ITS, internal transcribed spacer; 28S, large ribosomal subunit; OG, out-group;. Asterisk indicated the genetic data of samples obtained in the present study.

**Figure 5.**
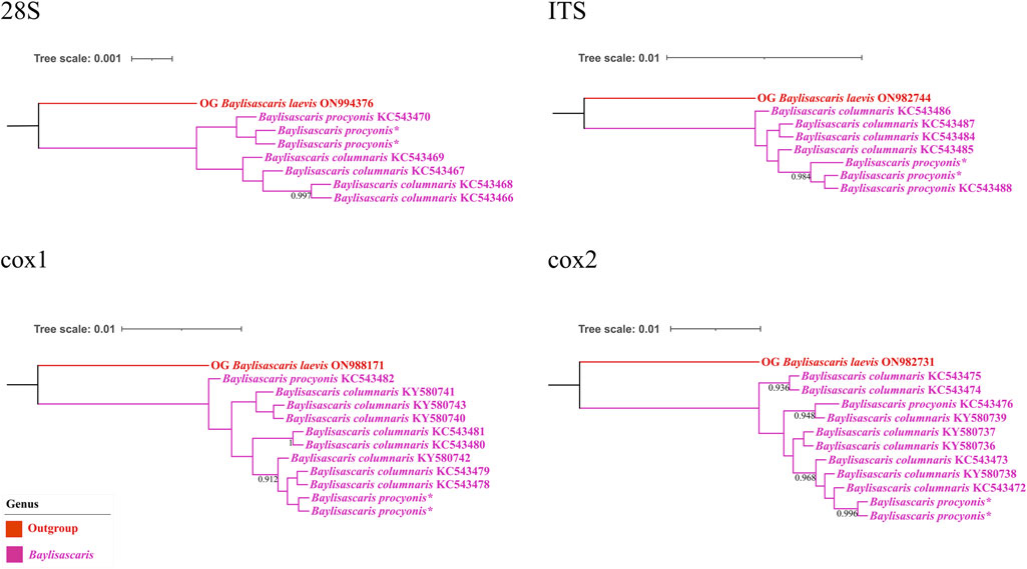
Bayesian inference analyses of *Baylisascaris procyonis* and *B. columnaris* based on 4 different nuclear and mitochondrial genetic markers, respectively. Bayesian posterior probabilities values ≥0.70 were shown on nodes. Asterisk indicated the genetic data of samples obtained in the present study.

Molecular analysis of *B. procyonis* and *B. columnaris* revealed the presence of a very low level of nucleotide divergence between the 2 species in the 28S, ITS, *cox*1 and *cox*2 regions (Figures 4, 5; please see Tables 4–7 for the details). ASAP analyses of *B. procyonis* and *B. columnaris* using the 28S, ITS, *cox*1, and *cox*2 sequence data all did not support the current species partition of these 2 species (Fig. 4). Bayesian inference analyses based on the ITS, *cox*1, and *cox*2 sequence data also showed samples of *B. procyonis* mixed with *B. columnaris* (Fig. 5), which are accordant with the ASAP results. Although the result of BI analysis based on the 28S sequence data displayed *B. procyonis* and *B. columnaris* formed 2 distinct clades with weak support, the present molecular analysis revealed the presence only 1 polymorphic loci between the partial 28S region of these 2 species (Table 4).

**Table 4.** Base difference in the partial 28S region between *B. columnaris* and *B. procyonis*

Species	28S fragment (717 bp)	
	49	487
B. procyonis*_OR457646	T	C
B. procyonis*_OR457647	T	C
*B. procyonis*_KC543470	T	C
*B. columnaris*_KC543466	C	G
*B. columnaris*_KC543467	C	C
*B. columnaris*_KC543468	C	G
*B. columnaris*_KC5434869	C	C

Asterisks indicate the sequences obtained herein.

**Table 5.** Base difference in the partial ITS region between *B. columnaris* and *B. procyonis*

Species	ITS fragment (651 bp)								
	158	201	271	540	541	542	543	544	545
B. procyonis*_OR453333	–	C	A	A	G	A	G	A	G
B. procyonis*_OR453334	–	C	G	A	G	A	G	A	G
*B. procyonis*_KC543488	–	C	G	A	G	A	G	A	G
*B. columnaris*_KC543484	–	T	G	–	–	–	–	–	–
*B. columnaris*_*KC543485*	A	T	G	A	G	–	–	–	–
*B. columnaris*_KC543486	–	T	G	–	–	–	–	–	–
*B. columnaris*_KC5434847	A	T	G	A	G	–	–	–	–

Asterisks indicate the sequences obtained herein.

**Table 6.** Base difference in the partial *cox*1 region between *B. columnaris* and *B. procyonis*

Species	*cox*1 fragment (413 bp)						
	59	61	79	91	229	259	353
B. procyonis*_OR453235	C	G	T	A	A	G	T
B. procyonis*_OR453236	C	G	T	A	A	G	T
*B. procyonis*_KC543476	T	A	T	G	A	A	G
*B. columnaris*_KC543472	C	G	T	A	A	G	G
*B. columnaris*_KC543473	C	G	T	A	A	G	G
*B. columnaris*_KC543474	T	G	C	A	G	G	G
*B. columnaris*_KC543475	T	G	C	A	G	G	G
*B. columnaris*_KY580736	T	G	T	A	A	G	G
*B. columnaris*_KY580737	T	G	T	A	A	G	G
*B. columnaris*_KY580738	C	G	T	A	A	G	G
*B. columnaris*_KY580739	T	A	T	G	A	G	G

Asterisks indicate the sequences obtained herein.

**Table 7.** Base difference in the partial *cox*2 region between *B. columnaris* and *B. procyonis*

Species	*cox*2 fragment (483 bp)				
	66	117	246	280	480
B. procyonis*_OR463271	G	A	T	C	G
B. procyonis*_OR463272	G	A	T	C	G
*B. procyonis*_KC543482	A	A	T	T	G
*B. columnaris*_KC543478	G	A	T	C	G
*B. columnaris*_KC543479	G	A	T	C	G
*B. columnaris*_KC543480	G	G	T	T	T
*B. columnaris*_KC543481	G	G	T	T	T
*B. columnaris*_KY580740	G	A	C	T	G
*B. columnaris*_KY580741	A	A	C	T	G
*B. columnaris*_KY580742	G	A	T	C	G
*B. columnaris*_KY580743	G	A	C	T	G

Asterisks indicate the sequences obtained herein.

### Phylogenetic analyses of *Baylisascaris* spp.

**Figure 6.**
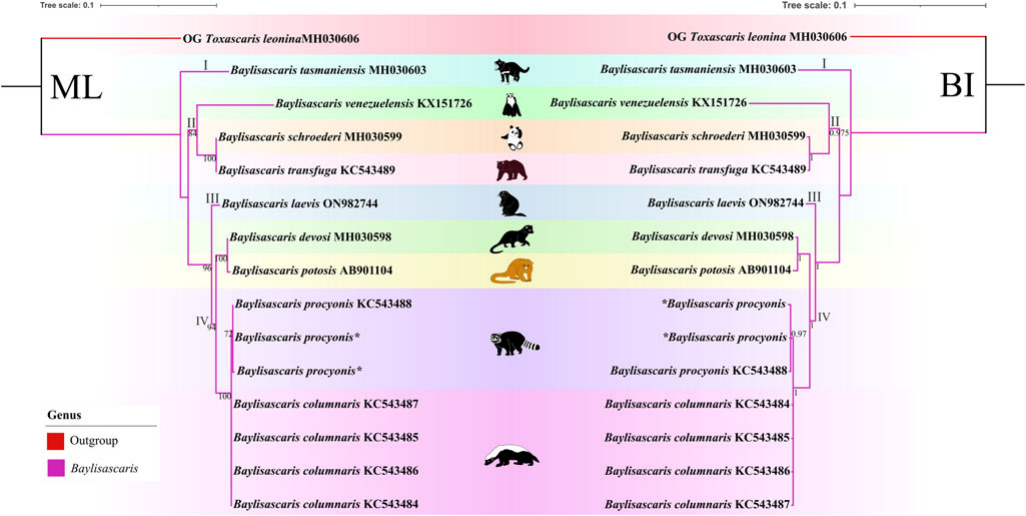
Phylogenetic relationships of representatives of the *Baylisascaris* using maximum likelihood and Bayesian inference analyses based on the ITS sequences. *Toxascaris leonina* (Ascaridomorpha: Ascarididae) was chosen as the out-group. Bootstrap values ≥70 and Bayesian posterior probabilities values ≥0.70 were shown on nodes in the phylogenetic trees. Asterisk indicated the genetic data of samples obtained in the present study.

**Figure 7.**
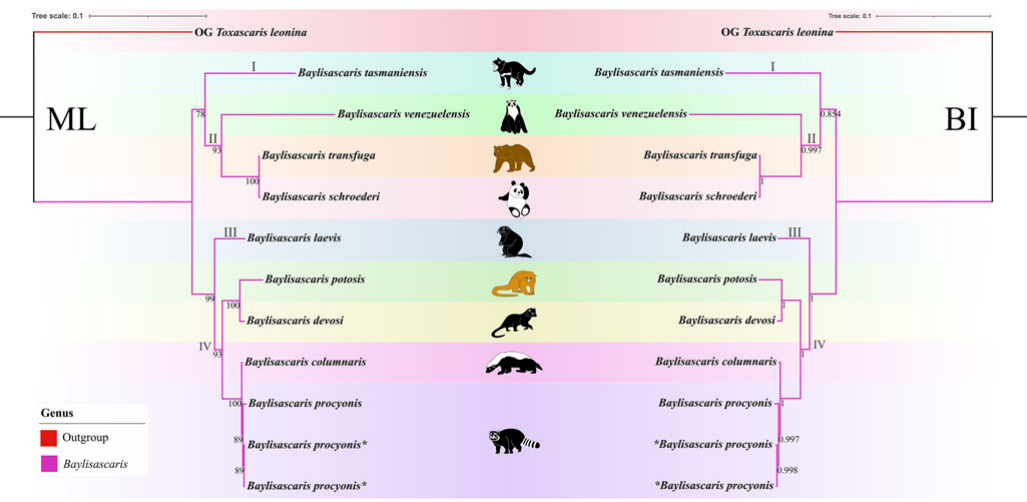
Phylogenetic relationships of representatives of the *Baylisascaris* using maximum likelihood and Bayesian inference analyses based on the ITS + 28S + *cox*1 + *cox*2 sequences. *Toxascaris leonina* (Ascaridomorpha: Ascarididae) was chosen as the out-group. Bootstrap values ≥70 and Bayesian posterior probabilities values ≥0.70 were shown on nodes in the phylogenetic trees. Asterisk indicated the genetic data of samples obtained in the present study.

(Figures 6, 7). The results of phylogenetic analyses based on the ITS sequence data using ML and BI methods were more or less identical in topology, which displayed species of *Baylisascaris* divided into 4 clades (clade I, II, III and IV) (Fig. 6). *Baylisascaris tasmaniensis* located at the base of the phylogenetic trees representing clade I, which formed a sister relationship with the other species of *Baylisascaris*. Clade II included *B. transfuga*, *B. schroederi* and *B. venezuelensis*, all parasitic in the ursid hosts. Among them, *B. transfuga* and *B. schroederi* showed closer relationship than *B. venezuelensis*. Clade III contained only *B. laevis* reported from the rodent definitive hosts. Clade IV comprising *B. devosi*, *B. potosis*, *B. columnaris* and *B. procyonis*, parasitising the mustelid and procyonid hosts. Among them, *B. procyonis* clustered together with *B. columnaris*, and *B. devosi* is a sister to *B. potosis*.

Phylogenetic results based on the 28S + ITS + *cox*1 + *cox*2 sequence data using ML and BI methods were slightly different from the phylogenetic results based on the ITS data, which showed *B. tasmaniensis* is a sister to *B. transfuga* + *B. schroederi* + *B. venezuelensis* (Fig. 7). Phylogenetic relationships of these species *B. laevis*, *B. devosi*, *B. potosis*, *B. procyonis* and *B. columnaris* agreed well with that of the phylogenetic trees based on the ITS data (Fig. 7).

## Discussion

Stefanski and Zarnowski (1951) described *Ascaris procyonis* from the intestine of *P. lotor* from a zoo in Poland. Sprent (1968) transferred this species to the genus *Baylisascaris*. Although *B. procyonis* has been studied extensively with respect to development and life cycle (Kazacos, 1983; Donnelly *et al.*, 1989; Sakla *et al.*, 1989), population genetic structure (Blizzard *et al.*, 2010; Al-Warid *et al.*, 2018), mitochondrial genome (Xie *et al.*, 2011*a*, 2011*b*), prevalence and epidemiology of host (Snyder and Fitzgerald, 1987; Matoba *et al.*, 2006; Kresta *et al.*, 2010; Hernandez *et al.*, 2013; Jardine *et al.*, 2014; Page *et al.*, 2016; Al-Warid *et al.*, 2017; Thornton *et al.*, 2020), human infection and pathologies (Fox *et al.*, 1985; Murray, 2002; Gavin *et al.*, 2005; Hung *et al.*, 2012; Bauer, 2013); the morphology of this species has received little attention since its original description, and only the compilations of Hartwich (1962), Sprent (1968), Overstreet (1970), Kazacos and Turek (1982) and Snyder (1989) included it.

The morphology and morphometric data of the present specimens more or less agreed with the previous descriptions of *B. procyonis* by Stefanski and Zarnowski (1951), Hartwich (1962), Sprent (1968) and Overstreet (1970), including the lengths of body and oesophagus, the morphology of lips and lateral alae, the morphology and length of spicules, the number and arrangement of precloacal papillae, the morphology of cloacal ornamentations, the relative position of vulva, the size of eggs, and the length of tail (See Table 1 for details). In addition,

our specimens are also collected from the type of host *P. lotor*. Consequently, we considered our specimens to be *B. procyonis*. However, the length of spicules in our specimens is slightly shorter than that in some previous descriptions (Stefanski and Zarnowski, 1951; Hartwich, 1962; Sprent, 1968), but it is accordant with Overstreet's (1970) description. Moreover, Stefanski and Zarnowski (1951) and Overstreet (1970) both considered the tail of male with small spike-like tip. However, we did not observe that in our specimens using s.e.m.

The morphology of *B. procyonis* is very similar to *B. columnaris*, and some previous phylogenetic studies also supported *B. procyonis* and *B. columnaris* have a close affinity (Franssen *et al.*, 2013; Tokiwa *et al.*, 2014; Mata *et al.*, 2016; Camp *et al.*, 2018; Sharifdini *et al.*, 2021). Overstreet (1970) considered the morphology of lips and first pair of postcloacal papillae to be important characters for distinguishing *B. procyonis* from *B. columnaris* (lips possessing remarkable medio-apical notch and the first pair of postcloacal papillae being double in *B. procyonis vs* the medio-apical notch of lips unconspicuous and the first pair of postcloacal papillae not being double in *B. columnaris*). The present s.e.m. observations confirmed the presence of small obtusely triangular medio-apical notch on each lip and first pair of postcloacal papillae being double in *B. procyonis*. However, the s.e.m. observations of *B. procyonis* by Kazacos and Turek (1982) found the absence of medio-apical notch on the lips of their specimens from *P. lotor*. Kikuchi and Oshima (1977) observed the detailed morphology of *B. columnaris* based on specimens collected from a skunk using s.e.m., and revealed the presence of small obtusely triangular medio-apical notch on the lips. Additionally, the number and morphology of postcloacal papillae of ascaridoid nematodes often vary between different individuals collected from a same or different hosts, even between the rows of postcloacal papillae in single individual (Uni and Takada, 1981; Li *et al.*, 2016; Zhao *et al.*, 2017). For example, some previous studies reported the presence of 2 closely associated single papillae instead of the postcloacal double papillae in their material of *B. transfuga* (Baylis and Daubney, 1922; Okoshi *et al.*, 1961; Tenora *et al.*, 1989). Snyder (1989) also pointed out that the first pair of postcloacal papillae of some of his specimens of *B. procyonis* was not double papillae and appeared as 2 single closely associated papillae based on s.e.m. observations. Moreover, Kikuchi and Oshima (1977) confirmed the first pair of postcloacal papillae of *B. columnaris* was double papillae in their material. Consequently, it is not reasonable and reliable to use the absence or presence of medio-apical notch on lip and the morphology of first pair of postcloacal papillae (single or double) as criteria for delimitation of *B. procyonis* and *B. columnaris*.

Franssen *et al.* (2013) and Choi *et al.* (2017) reported some loci of nucleotide polymorphisms in both mitochondrial (i.e. *cox1*, *cox2*, *ND2*, and several tRNA genes) and nuclear markers (ITS) and considered that these loci of nucleotide polymorphisms could be used as a tool to differentiate *B. procyonis* from *B. columnaris*. However, the present results of ASAP and BI analyses do not support that *B. procyonis* and *B. columnaris* represent 2 distinct species, and indicated that these loci of nucleotide polymorphisms in the 28S, ITS, *cox*1, and *cox*2 regions do not represent fixed differences between *B. procyonis* and *B. columnaris*. The validity of the important zoonotic species *B. procyonis* previously supported by morphological features (Hartwich, 1962; Sprent, 1968; Overstreet, 1970) and molecular data (Franssen *et al.*, 2013; Choi *et al.*, 2017) was challenged by the present study. Our results are consistent with the previous study (Camp *et al.*, 2018). Due to the current morphological studies and genetic analyses of *B. procyonis* and *B. columnaris*, it is possibly premature to treat *B. procyonis* as a synonym of *B. columnaris*. A more rigorous study integrating compared morphological study and molecular analyses with broader samples of *B. procyonis* and *B. columnaris* collected from different localities and hosts worldwide is required to solve the taxonomical status of these 2 species.

Among the 9 species of *Baylisascaris*, *B. tasmaniensis* showed sister relationship to the remaining *Baylisascaris* in the phylogenetic trees based on the ITS data, which are accordant with the previous phylogenetic studies (Sharifdini *et al.*, 2021; Barrera *et al.*, 2022), but are in conflict with other phylogeny (Camp *et al.*, 2018). The phylogenetic relationships of *B. tasmaniensis* and the others *Baylisascaris* spp. can be easily understood, because *B. tasmaniensis* only parasitises the marsupial carnivores in Australia (i.e. *Sarcophilus harrisii*, *Dasyurus viverrinus* and *Dasyurops maculatus*) and possesses some particular morphological features (i.e. the presence of fan-shaped lips, 3 pairs of postcloacal double papillae and slender spicules) (Sprent, 1970). Phylogenetic construction based on the ITS and 28S + ITS + *cox*1 + *cox*2 sequence data all supported these 3 species *B. transfuga*, *B. schroederi* and *B. venezuelensis* all parasitic in the ursid hosts, have a close affinity, that is identical to the previous studies (Mata *et al.*, 2016; Sharifdini *et al.*, 2021). Barrera *et al.* (2022) showed the systematic status of *B. laevis* based on molecular phylogeny for the first time. *Baylisascaris laevis* is the only known species in this genus to parasitise the rodent definitive hosts, e.g. marmot and ground squirrel (Rodentia: Sciuridae) (Sprent, 1968; Barrera *et al.*, 2022), which showed sister relationship with the 4 *Baylisascaris* species (*B. devosi* + *B. potosis* + *B. procyonis* + *B. columnaris*) parasitic in the carnivorous definitive hosts including mustelids and procyonids. Our phylogenetic results also revealed *B. devosi* is a sister to *B. potosis* for the first time. However, *B. procyonis* and *B. potosis* both hosted the procyonids, did not display a close relationship (the similar situation also occurring in *B. devosi* and *B. columnaris*), that possibly indicated that it is not reasonable to use the specific groups of definitive hosts (Procyonidae or Mustelidae) as a criterion for distinguishing *procyonis* from *B. columnaris*.

The present study revealed some previously unreported morphological features of *B. procyonis*. The results of morphological study and ASAP and BI analyses all did not support that *B. procyonis* and *B. columnaris* represent 2 distinct species and the validity of *B. procyonis* was challenged. Molecular phylogeny provided new insights into the evolutionary relationships of *Baylisascaris* spp.

## Data Availability

The nuclear and mitochondrial DNA sequences of *Baylisascaris procyonis* obtained in the present study were deposited in GenBank database [sequences of *B. procyonis* under the accession numbers: OR457646, OR457647 (28S), OR453333, OR453334 (ITS), OR453235, OR453236 (*cox*1), OR463271, OR463272 (*cox*2)]. Voucher specimens of *B. procyonis* were deposited in the College of Life Sciences, Hebei Normal University, Hebei Province, China (HBNU-N-2023M006G-L).
